# Diarrheal disease and associated behavioural factors among food handlers in Addis Ababa, Ethiopia

**DOI:** 10.3934/publichealth.2020010

**Published:** 2020-02-18

**Authors:** Aderajew Mekonnen Girmay, Sirak Robele Gari, Bezatu Mengistie Alemu, Martin R. Evans, Azage Gebreyohannes Gebremariam

**Affiliations:** 1 Ethiopian Institute of Water Resources, Addis Ababa University, Addis Ababa, Ethiopia; 2 College of Health and Medical sciences, Haramaya University, Harar, Ethiopia; 3 Microbiology Consultant and Laboratory Director, New York, USA

**Keywords:** diarrheal disease, food handler, behavioural factor, Addis Ababa, Ethiopia

## Abstract

Introduction: Diarrheal diseases are threat everywhere, but its frequency and impact are more severe in developing countries. Diarrhea occurs world-wide and causes 4% of all deaths and 5% of health loss to disability. In 2016, it was the eighth leading cause of mortality. Moreover, data from the World Health Organization indicated that diarrheal diseases are causes for an estimated 2 million deaths annually. Therefore, this study aimed to assess diarrheal diseases and associated behavioural factors. Method: An institution based cross-sectional study was conducted. A stratified random sampling method was employed to select 1050 study participants. Participants were interviewed using structured questionnaire. To analysis the data, binary logistic regression and multivariable logistic regression analysis was conducted. Results: The two weeks prevalence of diarrhea was found to be 3.4%. Further, 1.6%, 10.5%, 10.7% and 9% of the food handlers had acute watery diarrhea, cough, an infection of runny nose and incidence of any fever respectively. Regular hand washing after toilet (AOR = 0.13 with 95% CI: 0.024, 0.72), using toilet while wearing protective clothes/gown (AOR = 5.39 with 95% CI; 1.59, 18.32), habit of eating raw beef and raw vegetables (AOR = 6.27 with 95% CI: 1.89–20.78), type of toilet (AOR = 4.07 with 95% CI: 0.29–6.67 were associated significantly with diarrhea. Conclusion: This assessment proved to be an essential activity for reduction of community diarrheal diseases, as a significant number of food handlers had diarrhea. Good sanitation, hygiene practice and a healthy lifestyle behavior can prevent diarrhea. A strong political commitment with appropriate budgetary allocation is essential for the control of diarrheal diseases.

## 1. Introduction

Diarrhea is defined as three or more loose or watery stools per day [1]. Diarrhea is a threat everywhere, but its frequency and impact are more severe in low-resource settings [2],[3]. It occurs world-wide and causes 4% of all deaths and 5% of health loss to disability [4]. In 2016, diarrheal diseases were the eighth leading cause of death among all ages [5]. Diarrheal diseases caused an estimated 1.3 million deaths and are the fourth leading cause of years of life lost in developing countries [6]. On a global scale, of the estimated 165 million *Shigella* diarrheal episodes estimated to occur each year, 99% occur in developing countries [7]. The major six pathogens responsible for diarrhea are *Shigella*, rotavirus, adenovirus, enterotoxigenic *Escherichia coli* *(E. coli) Cryptosporidium*, and *Campylobacter*. However, rotavirus and *E. coli* are the two most common etiological agents of moderate-to-severe diarrhea in low-income countries [8].

Diarrheal disease affects rich and poor, old and young, and those in developed and developing countries alike, yet a strong relationship exists between poverty and an unhygienic environment. Food-borne diseases pose a significant public health burden worldwide [9],[10]. Diarrheal diseases are the most common illnesses resulting from the consumption of contaminated food and water [11],[12]. In Africa, it is estimated that 92 million people fall ill from consuming contaminated foods, resulting in 137,000 deaths each year [13]. In developing countries, there have been several attempts to improve food safety and to reduce diarrheal disease [14]. However, insufficient access to adequate hygiene and sanitation are major risk factors for the heavy burden of diarrheal diseases in developing countries [15]. Biological contaminants, largely bacteria and parasites constitute the major causes of diarrheal diseases often transmitted through food, water, and nails, and fingers contaminated with faeces. Accordingly, food handlers with poor personal hygiene are potential sources of infections from these microorganisms [16]. Based on the distribution of use of the different types of water sources and the associated risks of diarrhea, 502,000 diarrheal deaths in low and middle income countries can be attributed to inadequate and unsafe drinking water. Of these deaths, 88% occur in Africa and South-East Asia [17]. Moreover, in 2016, impure water, inadequate sanitation and poor hygiene was responsible for 829,000 annual deaths from diarrhea, and 1.9% of the global burden of disease [18].

Though the federal government of Ethiopia launched an urban health extension program including food and water safety packages at the capital city, Addis Ababa in 2009, still the city has many health problems especially recurrent food and water borne outbreaks [19]. According to the 2016 Addis Ababa health bureau report, there is high prevalence of diarrheal diseases in Addis Ababa though its sources are not well known and studied in depth. According to the 2017 Addis Ababa Food, Medicine and Health Care Administration and Control Authority (AAFMHACA) Report, food establishments located in Addis Ababa are suspected to be major sources of diarrheal diseases which might arise from poor knowledge and practice of food handlers, poor quality of drinking water, poor waste water and solid waste management, lack of wash facilities and poor water storage conditions. Due to these and other unknown factors, the city has many health problems like a high burden of typhoid fever, amoeba, acute watery diarrhea and other diarrheal diseases. Diarrheal disease is an important public health problem, causing morbidity and mortality. However, except for verbal reports, no studies have been conducted on the health status of food handlers. Because of this, a considerable number of customers of the food establishments have been exposed to different gastro-intestinal health problems. Accordingly, this study aimed at filling the research gap on the prevalence of diarrheal disease and associated behavioral factors among food handlers in Addis Ababa.

## 2. Methods

### 2.1. Description of the study area

**Figure 1.**
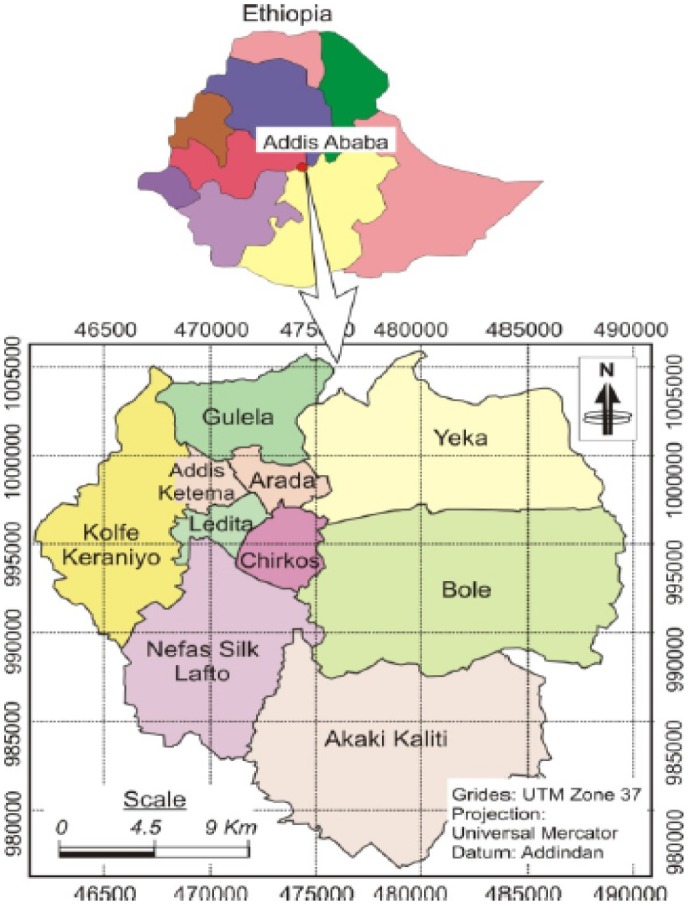
Map of Addis Ababa city administration. Source: Berhanu M, Raghuvanshi TK, Suryabhagavan K, Web-based GIS approach for tourism development in Addis Ababa city, Ethiopia [20].

The study was conducted in Addis Ababa city located in Upper Awash River basin, the capital of the Federal Government of Ethiopia and the seat for the African Union Headquarters. According to the 2017 AAFMHACA report, there are 1141 licensed food establishments, employing 4565 food handlers. Of the total licensed food establishments, 95 (8%) are large (hotels with one or more stars) and the remaining 1046 (92%) are small food establishments which include unranked (non-star hotels, bars, restaurants, cafes etc). The location map of Addis Ababa city is depicted below in Figure 1.

### 2.2. Study design

An institutional based cross sectional study was conducted among food handlers of Addis Ababa city administration from June to July 2019.

### 2.3. Source population

All food handlers located in Addis Ababa city administration were the source population.

### 2.4. Study population

All selected food handlers were located in Addis Ababa.

### 2.5. Inclusion and exclusion criteria

All 18 years and above food handlers, workings for a minimum of three months were included. However, causal food handlers were excluded.

### 2.6. Sample size determinations

To estimate the two week prevalence of diarrheal diseases and associated behavioral factors among food handlers in the food establishments of Addis Ababa, a sample size was calculated using a two population proportion formula (EPI INFO version 7.2.2.6) considering that:

For hand washing before meals and after defecation:

- P1 prevalence of diarrhea among exposed group is 16.9% and P2 prevalence of diarrhea among unexposed group is 24.1% [21].
- Power to detect a significant difference between P1 and P2 if it exists (1 − β) = 80%.
- Z α/2 = 95% confidence interval.
- Ratio [r] = 1:1 and OR= 0.643 [21].
- The weighted average of P1 and P2 = $\text{P}=\frac{\text{P}1+\text{r}(\text{P}2)}{1+r}=\frac{0.893+1(0.107)}{1+1}=0.5$

n = sample size for hand washing before meals and after defecation.

Then, using two population proportion formula, 
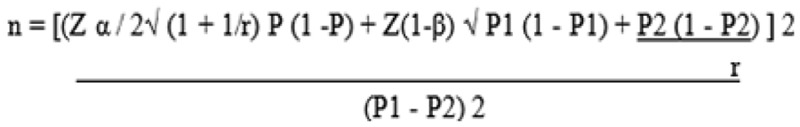
(1)

Or EPI INFO versions 7.2, 2.6 STATCALC, 1058 sample of food handlers were included.

### 2.7. Sampling procedure

**Figure 2.**
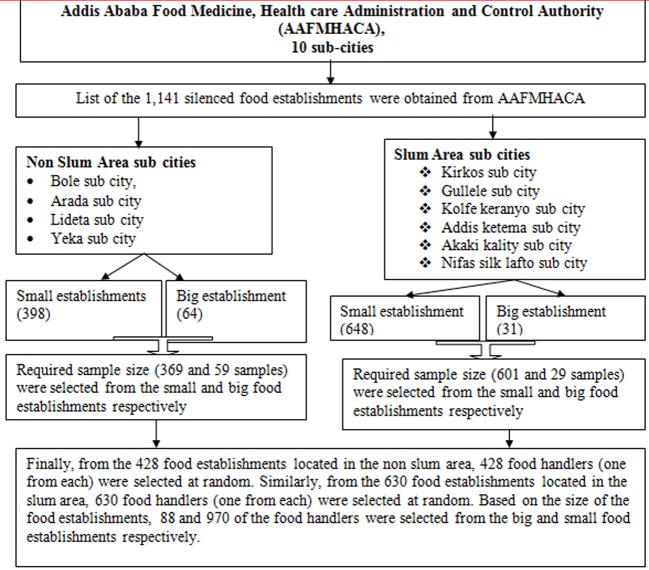
Systematic structure of the study sampling procedure.

The study participants were selected using a stratified, simple random sampling technique. To collect data, a listing of the 1141 licensed food establishments was obtained from AAFMHACA. These 1141 food establishments were stratified in to slum and non-slum areas based on their location. Similarly, the food handlers were stratified into two based on their work location (slum and non-slum area). Further, food handlers working in large and small food establishments were stratified into two. Based on this, sample allocation was conducted to the slum and non-slum areas in addition to the large and small food establishments. After the food handlers were stratified based on their work location and size of food establishments (large or small), one food handler from one food establishment was selected at random. Based on this, samples from 428 and 630 food handlers were taken from the food establishments located in the non-slum and slum area respectively. From the non-slum area, 59 samples from the large/big and 369 samples from the small food establishments were collected. Also, from the slum area (630), 29 samples from the large/big and 601 samples from the small food establishments were collected. Finally, using a simple random sampling technique, 1058 food handlers were selected to assess prevalence of diarrheal diseases and associated factors. A stratified random sampling technique was used in both slum and non-slum areas as well as large and small food establishments of Addis Ababa. The main purpose of stratification was to ensure representativeness for food handlers working in different areas and in various types of establishments with different educational status and work experience. In summary, the sampling procedure for this study is depicted below in Figure 2.

### 2.8. Data collection procedures

Data enumerators were identified based on professional capability and technical experience in collecting the required data. Accordingly, fifteen health professionals with a Bachelor of Science and extensive experience in similar data collection practices were employed. In addition, four Masters' degree holders were recruited for supervision of data collection. Two-day training was given to the data collectors and supervisors. After written consent was obtained from each study subjects, the data was collected from food handlers through face-to-face interview using a structured questionnaire.

### 2.9. Data quality assurance

A questionnaire was prepared in English and translated in to Amharic and back to English to maintain the consistency of questions. The quality of data was ensured through training of data collectors, close supervision, prompt feedback and daily recheck of completed questionnaire. Moreover, a brief daily activity evaluation method was established to correct problems that arose during the course of data collection. The consent and the assurance of confidentiality were ensured. The principal investigator checked and reviewed the entire completed questionnaire to ensure completeness and consistency of the information.

### 2.10. Data analysis

All data were checked for correctness of information and code. Date analyses were performed by using SPSS (Statistical Package for the Social Sciences) software version 20. Descriptive statistics such as frequency (%) for categorical and mean and standard deviation for numerical data were used to sum up the data. A binary logistic regression model was fitted to assess any association between diarrhea and independent variables. P values of 0.05 and 95% confidence interval for adjusted odds ratio (AOR) were used to report statistical significance.

### 2.11. Operational definitions of key terms

Diarrhoea Disease: Defecation frequency of three or more loose/liquid stools in a day.

Health Status: The presence of absence of diarrheal disease in two weeks prior to the study.

Food Establishments: Institutions that provide food and drinks for selling to customers.

Food: A material consisting nutritious substances that people eat or drink in order to maintain life and growth

Food Handlers: A person who is involved in the preparation and handling of food in a food establishment.

Large/big Food Establishment: Hotels with one or more stars.

Small Food Establishment: Small vendors, non-star hotels, bars, restaurants, cafes.

Slum Area: Area with poorer sanitation infrastructure.

Non-slum Area: Area with better sanitation infrastructure.

### 2.12. Study variables

A: Independent or explanatory variables:

The predictor variables of this study were sex, age, marital status, religion, educational status, length of work experience of the food handlers etc.

B: Dependent or outcome or response variables:

Outcome of this study was diarrheal disease.

### 2.13. Ethical consideration

Firstly, a letter of support was obtained from the Ethiopian Institution of Water Resources, Addis Ababa University. Then, ethical approval was obtained from the Ethiopian Public Health Institute Scientific and Ethical Review Board with reference number EPHI 613/138 in June 2019. To collect the data, written consent was obtained from each respondent after the objectives of this particular study were explained. Candidates were informed that their participation was voluntary. Confidentiality and privacy of respondents were ensured throughout the research process. The study design did not harm those taking part and it did not include any identifying information like name, or address of respondents.. They were well informed by the data collectors that the study was only for the purpose of academic and institutional research and not for any other business or illegal activities. Then, data were collected after assuring the confidential nature of responses.

## 3. Results

### 3.1. Socio-demographic characteristics of the food handlers

A total of 1050 food handlers participated in the study with a response rate of 99.2%. In the current study, 77% of the participants were female. Of the total participants, 43.4% and 28.7% were between the age group of 18 to 22 and 23 to 27 years old respectively. The mean age of the respondents was 25.695 years. 82.7% of the food handlers had the ability to read and write, while 17.3% were illiterate. The majority of the participants (81.2%) were single. Of the total respondents, 73% of participants were Orthodox Christians and 13.1% were Muslims. Regarding work experience, 46.3% and 35.3% of the respondents had 1 to 5 years and <1 year of work experience as food handlers respectively. Furthermore, 17.8% of the food handlers had above 5 years of work experience as food handlers. The average length of food handlers work experience was found to be 3.34 years (Table 1).

**Table 1.** Socio-demographic characteristics of the food handlers (n = 1050).

Study variables	Category	Frequency	Percent
Sex of the food handlers	Male	242	23.0
	Female	808	77.0
Age group of the food handlers	18–22 years	456	43.4
	23–27 years	301	28.7
	28–32 years	127	12.1
	>32 years	166	15.8
Mean age of the food handles	25.695 years with SD of ±7.576		
Minimum age of the food handlers	18.00 years		
Maximum age of the participants	65.00 years		
Educational status of the food handlers	Illiterate	182	17.3
	At least read and write	868	82.7
Marital status of the food handlers	Single	853	81.2
	Married	180	17.1
	Divorced and others	17	1.6
Religion of the food handlers	Orthodox	766	73.0
	Muslim	138	13.1
	Others	146	13.9
Work experience of the food handlers	<1 year	371	35.3
	1 to 5 years	492	46.9
	>5 years	187	17.8
Mean work experience of the food handlers	3.34 years with SD of ±3.37 years		

### 3.2. Food handlers work profile, medical checkup practice and training situation

The study found 45.1% and 44% of the food handlers' role was cooking and serving as waiters respectively. Of the total participants, 85% and 15% of the food handlers live and sleep in their home and at the food establishments respectively. More than half (57.4%) of the food handlers had no medical checkup or health examination certificate within the past three months prior to the study. From the total respondents, 61.3% of the food handlers reported that there was a mechanism of isolation for sick food handlers from the workplace. However, a greater number of food handlers (83.1%) had no training on food and water safety at least once in the past year prior to the study (Table 2).

### 3.3. Type of disease symptom and morbidity among the food handlers

Out of the 1,050 food handlers, 36 had diarrhea two weeks before the interview or a prevalence of 3.4%. Further, from the total participants, 17 (1.6%) of the food handlers had Acute Watery Diarrhea confirmed by a laboratory in the past year prior to this study. Moreover, 10.5%, 10.7% and 9% of the food handlers had a cough, infection or runny nose (influenza) and the incidence of fever within the past two weeks prior to this study respectively (Table 3).

**Table 2.** Food handlers work profile, medical checkup practice and training situation (n = 1050).

Study variables	Category	Frequency	Percent
Role of food handlers in the food establishment	Cook	474	45.1
	Waiter	462	44.0
	Both cooker and waiter	114	10.9
Place of food handlers living and sleeping	At the food establishment	158	15.0
	At her/his home	892	85.0
Type of employee in the food establishment	Permanent	177	16.9
	Temporary	873	83.1
Medical checkup or health examination certificate at least within every three month	Yes	447	42.6
	No	603	57.4
Isolation of sick food handlers from the work place when food handler is ill	Yes	644	61.3
	No	406	38.7
Training of food handles on food and water safety at least once in a year	Yes	177	16.9
	No	873	83.1

**Table 3.** Type of disease symptom and morbidity among the food handlers.

Study variables	Category	Frequency	Percent (%)
Diarrheal diseases within the past two weeks prior to this study	Yes	36	3.4
	No	1014	96.6
Acute Watery Diarrhea (AWD) confirmed by laboratory for the past one year prior to this study	Yes	17	1.6
	No	1033	98.4
Cough within the past two weeks prior to this study	Yes	110	10.5
	No	940	89.5
An infection of runny nose within the past two weeks prior to this study	Yes	112	10.7
	No	938	89.3
Incidence of any fever within the past two weeks prior to this study	Yes	95	9.0
	No	955	91.0

### 3.4. Factors that may be contribute to diarrheal diseases among food handlers

Of the total participants, 94.5% and 93.8% washed their hands regularly after using the toilet and before meal respectively. However, 39% of the respondents used the toilet wearing protective clothes/gown. Additionally, 81.9% of the food handlers washed their hands immediately after handling raw foods. Further, 91.6% and 92% of the participants regularly closed their drinking water container to prevent contamination, and regularly washed their drinking water container and utensils with sanitizers and disinfectants. Almost all (96.7%) of the participants were washed glasses or the materials used for drinking water at every event. Also, 82.3% of the respondents put cooked foods separately from raw foods. Although 91.1% of the respondents did not use the same chopping block and knife during processing raw food and cooked food, 24.1% of the participants had habits of eating raw beef and raw vegetables. The food handlers reported that 89% of them ate a meal regularly in the food establishments. However, only 33.3% of the participants used proper waste disposal methods. Further, 62.5% of the respondents utilized unimproved or traditional toilets (Table 4).

**Table 4.** Factors that may be contribute to diarrheal diseases among the food handlers.

Study variables	Category	Frequency	Percent
Regular hands washing after toilet used (defecation)	Yes	992	94.5
	No	58	5.5
Washing hands before meal regularly	Yes	985	93.8
	No	65	6.2
Used toilet while wearing protective clothes/gown	Yes	409	39.0
	No	641	61.0
Hand washing immediately after handling raw foods	Yes	860	81.9
	No	190	18.1
Take precaution/close drinking water container regularly	Yes	962	91.6
	No	88	8.4
Washing drinking water container and food service utensils with sanitizers and disinfectants regularly	Yes	966	92.0
	No	84	8.0
Washing glass or the material used for drink water every event with safe water	Yes	1015	96.7
	No	35	3.3
Put cooked foods separately from raw foods	Yes	864	82.3
	No	186	17.7
Habit of eating raw Beef and raw vegetables	Yes	253	24.1
	No	797	75.9
Used the same chopping block and knife during the time of processing raw food and cooked food	Yes	93	8.9
	No	957	91.1
Feed regularly in the food establishment	Yes	935	89.0
	No	115	11.0
Type of food establishment that the food handlers work	One and above one star Hotel	86	8.2
	Non star Hotel	69	6.6
	Bar and restaurant	194	18.5
	Cafe and restaurant	76	7.2
	Restaurant	424	40.4
	Cafe and others	201	19.1
Used proper waste disposal methods (pedal dust bin, septic tank)	Yes	350	33.3
	No	700	66.7
Type of toilet most of the time used by food handlers	Unimproved or traditional toilet	656	62.5
	Improved or water flush toilet	394	37.5
Presence of sanitary inspection by authorized bodies in the food establishment	Yes	865	82.4
	No	185	17.6
Type of the establishment in size the food handlers work	Small food establishment	964	91.8
	Big food establishment	86	8.2

### 3.5. Behavioral factors associated with diarrhea

In the binary logistic regression analysis, thirteen (13) explanatory variables like educational level of food handlers, regular hand washing after toilet used (defecation), regular hand washing before meal, used toilet with wearing protective clothes/gown, regular hand washing immediately after holding raw foods, closing drinking water container regularly, washing drinking water container with safe water and food service utensils with sanitizers and disinfectants, washing glass or the material used for drink water every event, separation of cooked foods from raw foods, habit of eaten raw beef and raw vegetables, used the same chopping block and knife during the time of processing raw food and cooked foods, type of toilet most of the time used by food handlers and presence of sanitary inspection by authorized bodies in the food establishment were significant associated (p-value < 0.028) with diarrheal disease in the past two weeks prior to this study. However, only five (5) predictor variables including: regular hand washing after toilet used (defecation), toilet use while wearing protective clothes/gown, washing glass or the material used for drink water every event, habit of eating raw beef and raw vegetables and type of toilet used by food handlers were appeared in the final condensed model of the multivariable analysis with P-value < 0.05 (Table 5).

**Table 5.** Multivariate logistic regression analysis of diarrheal disease with selected explanatory variables among the food handlers (n = 1050).

Study variables		Diarrhea		Β	Wald	P Value	AOR with 95%CI
		Yes	No				
Regular hand washing after toilet used or defecation	Yes	9	983	−2.029	5.496	0.019	0.13(0.024–0.72)
	No	27	31				Reference
Used toilet while wearing protective clothes/gown	Yes	28	381	1.684	7.283	0.007	5.39(1.59–18.32)
	No	8	633				Reference
Washing glass or the material used for drink water every event with safe water	Yes	11	1004	−4.724	15.532	0.000	0.009(0.001–0.093)
	No	25	10				Reference
Habit of eating raw Beef and raw vegetables	Yes	30	223	1.836	9.010	0.003	6.27(1.89–20.78)
	No	6	791				Reference
Improved or water flush Toilet used by food handler	Yes	7	387	1.405	4.079	0.043	Reference
	No	29	627				4.07(0.29–6.67)

## 4. Discussion

The aims of this study were to identify the prevalence of diarrheal disease and associated behavioral factors among food handlers. The self-reported prevalence of diarrheal disease in the two weeks before the interview was 3.4%.

This finding was lower than studies performed in Ethiopia and Haiti [22],[23]. This could be due to difference in attention given to health status and environmental risk factors. However, this result was consistent with a similar study conducted in Ireland [24]. This could be due to presence of good awareness among the food handlers towards diarrheal diseases. Further, this result was nearly consistent with a study conducted in South India where the prevalence of diarrhea among food handlers was 5.52% [25]. The slight difference might be due to the presents of recurrent food and water borne diseases in Addis Ababa and made alerted the food handlers about diarrheal diseases.

From the total participants, 17 (1.6%) of the food handlers had Acute Watery Diarrhea confirmed by laboratory testing in the past year prior to this study. Because we did not find information from literature on the prevalence of acute watery diarrhea among food handlers, this result needs further research because it is a major public health problem. Moreover, 10.5%, 10.7% and 9% of the food handlers had a cough, infection or runny nose (influenza) or the incidence of fever within the past two weeks prior to this study respectively. This indicates the health status of food handlers was poor though they were expected to be healthy and not transmit any infection to customers.

This study revealed that food handlers who had washed their hands after defecation or toilet use were 13% less likely to report diarrhea than those who did not report hand washing. This finding was supported by a study from low and middle income countries [26]. As expected, this may be due to removal of pathogenic organisms during proper hand washing after toilet use. Therefore, washing hands properly at the most recommended times is the key preventive mechanism of diarrheal disease. However, food handlers who used toilet while wearing protective clothes/gown had 5.39 times higher risk of diarrheal disease (AOR = 5.39 with 95% CI; 1.59, 18.32) relative to those who had not used the toilet while wearing personal protective device. This indicates personal protective equipment can carry pathogenic organisms or might be vehicles although no study reported this. Therefore, this needs future research to obtain additional information. Moreover, our finding revealed that food handlers who utilized washed glass or the material used for drinking water had prevented risk of diarrhea by 0.9% times higher (AOR = 0.009 with 95% CI: 0.001, 0.093) than those who did not. This indicates that, using safe water-washed glass reduces the risk of diarrheal disease. The odds of having diarrheal disease was 6.27 times higher among food handlers who had the habit of eating raw beef and raw vegetables (AOR = 6.27 with 95% CI: 1.89–20.78) than those who did not. This finding was supported by a 2017 study done in Bejing, China [21]. Further, the odds of having diarrheal disease was 4.07 times higher among those food handlers who used unimproved/traditional pit toilet (AOR = 4.07 with 95%CI: 0.29–6.67) than those who used improved or water flush toilet. Although in general the presence of a sanitary facility prevents different communicable diseases [26], this result shows using a traditional pit latrine had its own health impact on a community.

## 5. Conclusion

This study assessed the prevalence of diarrheal disease and identifies behavioral factors associated with diarrhea. This assessment proved to be an essential activity for reduction of community- acquired diarrheal diseases, as a significant number of food handlers had diarrhea. Good sanitation, hygiene practice and a healthy lifestyle behavior can prevent diarrhea. A strong political commitment with appropriate budgetary allocation is essential for the control of diarrheal diseases. The government should focus on a comprehensive diarrheal disease control strategy including improvement of water quality, hygiene, and sanitation. Current public health programs of the Addis Ababa city administration should develop effective approaches to promote hand washing practice and creation of awareness. Moreover, other interventions should be strengthened to reduce the occurrence of diarrhea. Improved interventions combined with formal training on food safety practice should be strengthened to reduce occurrence of diarrhea among the food handlers and to reduce health problems of their customers. Moreover, routine inspections should be conducted by authorized bodies to enhance hygiene and sanitation practices of food handlers.
